# Editorial: Remediation of Hazardous Materials in Gut by Probiotics and Intestine Microbiome

**DOI:** 10.3389/fnut.2022.941028

**Published:** 2022-07-06

**Authors:** Pu Liu, Saurabh Kulshrestha, Xiangkai Li

**Affiliations:** ^1^School of Life Sciences, Lanzhou University, Lanzhou, China; ^2^Faculty of Applied Sciences and Biotechnology, Shoolini University of Biotechnology and Management Sciences, Solan, India

**Keywords:** probiotics, microbiome, hazardous material, intestine, degradation

Gut microbiota, the vast microbial community that inhabits the gastrointestinal tract of humans and animals, plays vital roles in the maintenance of intestinal homeostasis and many other host processes. Over the last decade, there has been growing research interest in the structural and functional dynamics of gut microbiota exposed to various hazardous xenobiotics including both natural and synthetic compounds. Disruptions to the gut microbiota may cause subsequent alterations in metabolic and physiological processes, contributing to the progression of a wide range of diseases in the host. In this context, it has been hypothesized and supported by extensive studies that oral administration of probiotics can regulate the composition of intestinal microbiota and are effective in the bioremediation of toxic agents. Nevertheless, remediating hazardous materials by shaping gut microbiota requires a more thorough mechanistic understanding of the interaction networks between xenobiotics and gut microbes. Hence, in this Research Topic, both research and review papers have been collected to improve our knowledge and understanding of the function of gut microbiota in response to xenobiotics and the active mechanisms of probiotic strains to improve host health.

Probiotics are capable of surviving in the gastrointestinal tract after oral intake and reduce the xenobiotic residues by acting as binding agents. However, the effect of probiotics on shaping the gut microbiota to modulate/modify the dietary xenobiotic lifetime and bioavailability has not been studied extensively. Hence in this special e-collection, Hernández-Mendoza et al. give a comprehensive overview of the influence of probiotics on the functions of the gut microbiota to withstand and metabolize toxic agents, supporting the use of probiotics as regulators of intestinal microbiota.

Mycotoxins are one of the natural toxic xenobiotics that may cause adverse effects on human health. The study of Cai et al. shows in mice that a probiotic strain *Bacillus velezensis* A2 could alleviate cecal inflammation induced by Zearalenone, an estrogen-like mycotoxin, by regulating the composition of intestinal microflora and production of short-chain fatty acids by cecal microbes.

Gut microbiota and probiotics are well known to play roles in the nutrient metabolism of various hosts, for instance, in black soldier fly larvae (BSFL), *Hermetia illucens* (Diptera: Stratiomyidae), gut microbiota is key in converting organic waste into protein, and addition of probiotics can improve substrates conversion rate and protein content, making BSFL a promising alternative protein source. But less is known about the underlying mechanism of probiotics. Herein, the data from Pei et al. reported that a novel probiotic, *Bacillus velezensis* EEAM 10B (10B), significantly improved physiological parameters of BSFL, and the microbial composition of the gut was altered, with some species positively correlated with the physiological changes. The group further explored the function of 10B by analyzing the metabolism of germ-free and mono-bacterial intestinal BSFL and vitamins backfill assay and confirmed B-vitamin riboflavin provided by 10B was crucial to the survival of BSFL.

Immunotherapy by Immune Checkpoint Inhibitors (ICIs) is an important available treatment option against a variety of cancers and has been shown to provide therapeutic responses. It has also been observed that some non-responsive patients may exhibit hyperprogressive disease (HPD) as part of Immunotherapy. HPD subsequently leads to deterioration in the quality of life and also leads to a poor prognosis. The onset and mechanism of HPD are not being worked out properly and researchers are predicting it to be an outcome of multiple factors. Liu and Qiao, in their review article provided an updated overview of available information on HPD and also summarized the underlying mechanism of HPD.

In summary, the above manuscripts and reviews present some new relevant data on the *Probiotics and Intestine Microbiome*. Although studies on gut microbiota and xenobiotics interactions have accelerated in recent years, and the use of probiotics for the remediation of toxic agents appear to be a feasible approach in the area, the papers published in this e-collection show that there are still many aspects to be clarified. After reading this topic, readers will be clearer about the function of gut microbiota and probiotics on xenobiotics and hosts and be more convinced that the use of probiotics could open up a new way for protective and therapeutic approaches against hazardous agents.

## Author Contributions

PL and SK wrote the introduction and the conclusion of all articles published or accepted for publication in this special issue (SI). XL revised the manuscript critically. All authors contributed to the article and approved the submitted version.

## Conflict of Interest

The authors declare that the research was conducted in the absence of any commercial or financial relationships that could be construed as a potential conflict of interest.

## Publisher's Note

All claims expressed in this article are solely those of the authors and do not necessarily represent those of their affiliated organizations, or those of the publisher, the editors and the reviewers. Any product that may be evaluated in this article, or claim that may be made by its manufacturer, is not guaranteed or endorsed by the publisher.

